# Monitoring patients and asymptomatic carriers with hereditary transthyretin amyloidosis: regional protocol of Emilia-Romagna ATTR working group

**DOI:** 10.3389/fneur.2025.1666318

**Published:** 2025-08-29

**Authors:** Pietro Guaraldi, Isabella Allegri, Alessandra Ariatti, Tommaso Baldini, Andrea Barbieri, Federico Barocelli, Michela Bartolotti, Elena Biagini, Francesca Bianchi, Annamaria Borghi, Claudia Borghi, Giuseppe Boriani, Ilaria Cani, Samuela Carigi, Luca Codeluppi, Marco Currò Dossi, Francesca Dalpozzo, Roberto D’Angelo, Riccardo De Gennaro, Francesco Di Spigno, Elisa Gardini, Gianluca Lanati, Chiara Leuzzi, Francesca Marzo, Marco Masullo, Gaia Mazzanti, Elisa Merli, Agnese Milandri, Dennis Monari, Enrica Perugini, Alberto Ponziani, Emanuela Postiglione, Marta Rasia, Rita Rinaldi, Davide Scancarello, Kevin Serafini, Matteo Serenelli, Maurizio Sguazzotti, Ernesto Siena, Anna Maria Simone, Chiara Terracciano, Chiara Valenti, Giovanni Vitale, Maria Vitiello, Simone Longhi

**Affiliations:** ^1^IRCCS Istituto delle Scienze Neurologiche di Bologna, Bologna, Italy; ^2^Department of Neurology, University Hospital of Parma, Parma, Italy; ^3^Department of Biomedical, Metabolic Neural Sciences, University of Modena, Modena, Italy; ^4^Department of Neurology, Infermi Hospital, AUSL della Romagna, Rimini, Italy; ^5^Cardiology Division, Department of Biomedical, Metabolic and Neural Sciences, University of Modena and Reggio Emilia, Policlinico di Modena, Modena, Italy; ^**6**^Cardiology Division, Parma University Hospital, Parma, Italy; ^7^Cardiology Unit, Bufalini Hospital, AUSL della Romagna, Cesena, Italy; ^8^Cardiology Unit, Department of Cardiac Thoracic and Vascular, IRCCS Azienda Ospedaliero-Universitaria di Bologna, Bologna, Italy; ^9^European Reference Network for Rare, Low Prevalence and Complex Diseases of the Heart-ERN GUARD- Heart, Bologna, Italy; ^10^Neurology Unit, Bufalini Hospital Cesena, AUSL della Romagna, Cesena, Italy; ^11^IRCCS Istituto delle Scienze Neurologiche di Bologna, UOC Neurologia Rete Stroke Metropolitana Bologna, Bologna, Italy; ^12^Cardiology Unit, Ramazzini Hospital, Carpi, Italy; ^13^Cardiology Unit, Infermi Hospital, AUSL della Romagna, Rimini, Italy; ^14^Neurology Unit, Department of Neuromotor and Rehabilitation, Azienda USL- IRCCS di Reggio Emilia, Reggio Emilia, Italy; ^15^Neurology Unit, ‘Santa Maria delle Croci’ Hospital, AUSL della Romagna, Ravenna, Italy; ^16^Neurology Unit, Ospedale di Lugo, AUSL della Romgana, Lugo, Italy; ^17^Department of Neuroscience and Rehabilitation, Azienda Ospedaliero-Universitaria S, Anna, Ferrara, Italy; ^18^Cardiology Unit of Emergency Department, Guglielmo da Saliceto Hospital, Piacenza, Italy; ^19^Cardiology Unit, ‘Morgagni–Pierantoni’ Hospital, AUSL della Romagna, Forli, Italy; ^20^Cardiology Unit, Castel San Giovanni Hospital, Castel San Giovanni, Italy; ^21^Cardiology Unit, IRCCS Arcispedale S. Maria Nuova, Reggio Emilia, Italy; ^22^Cardiology Unit, Bellaria Hospital, IRCCS Istituto delle Scienze Neurologiche di Bologna, Bologna, Italy; ^23^Cardiology Unit, ‘Degli Infermi’ Hospital, AUSL della Romagna, Faenza, Italy; ^24^Cardiology Unit, Bentivoglio Hospital, Bologna, Italy; ^25^Cardiology Unit, Maggiore Hospital, Bologna, Italy; ^26^Cardiology Unit, Santa Maria della Scaletta Hospital, Imola, Italy; ^27^Department of Experimental, Diagnostic and Specialty Medicine, University of Bologna, Bologna, Italy; ^28^Cardiology Unit, Ospedale di Fidenza, Fidenza, Italy; ^29^Cardiology Unit, ‘Santa Maria delle Croci’ Hospital, AUSL della Romagna, Ravenna, Italy; ^30^Cardiology Unit, Azienda Ospedaliero Universitaria di Ferrara, Ferrara, Italy; ^31^Neurology Unit, Ospedale di Fidenza, Fidenza, Italy; ^32^Neurology Unit, Ramazzini Hospital, AUSL Modena, Carpi, Italy; ^33^Neurology Unit, 'Guglielmo da Saliceto' Hospital, Piacenza, Italy; ^34^Neurology and Stroke Unit, Department of Neuroscience, Bufalini Hospital, Cesena, Italy

**Keywords:** amyloid, transthyretin, multidisciplinary collaboration, standardized care, protocol, equity, equality, sustainability

## Abstract

Transthyretin amyloidosis (ATTR) is a rare disease caused by the extracellular accumulation of misfolded transthyretin (TTR) amyloid fibrils. ATTR can be either hereditary (ATTRv) or acquired (wtATTR). ATTRv is caused by a mutation in the transthyretin gene (TTR) with an autosomal dominant pattern of inheritance. In ATTRv amyloidosis, some patients primarily exhibit symptoms of polyneuropathy others mainly or exclusively present with symptoms of cardiomyopathy. However, many patients present with a multisystemic involvement that includes sensory, motor, autonomic, and cardiac symptoms. Early diagnosis and detection of disease progression are emerging as a crucial need for ATTR amyloidosis in order to significantly impact survival, patients’ functions and quality of life. Currently, parameters to be monitored in ATTR patients in the real life might refer to some publicly available recommendations regarding the monitoring and assessment of disease progression in the real-world setting of patients with ATTRv. Nonetheless, a standardized disease monitoring protocol has not been established in Italy, posing a significant unmet need for a prompt and equal access to care. Therefore, in the Emilia-Romagna Region the “ATTR Working Group” has sought to tailor the recommendations to the Regional “real clinical setting” in order to optimize and standardize a monitoring protocol aimed at identifying disease progression. Patients’ and carriers’ access to uniform monitoring routes across the entire Region ensures optimal disease management and economic sustainability.

## Introduction

Transthyretin amyloidosis (ATTR) is a rare disease caused by the extracellular accumulation of misfolded transthyretin (TTR) amyloid fibrils. This accumulation can affect multiple sites, including the nerves, heart and gastrointestinal tract. ATTR can be either hereditary (ATTRv) or acquired (wtATTR). ATTRv is caused by a mutation in the transthyretin gene (*TTR*) with an autosomal dominant pattern of inheritance. Wild-type transthyretin amyloidosis (wtATTR) is an age-related pathological condition due to TTR protein tetramer instability which leads to amyloid fibrils deposition mainly in the heart (1, 2). The prevalence of wtATTR in the elderly population is estimated to be around 10–25% in individuals over the age of 80. However, it is plausible that wtATTR amyloidosis is actually underdiagnosed (3).

A recent national survey revealed a 50% increase in diagnosed ATTRv cases in Italy over a 4-year period, with a shift towards p.Ile88Leu and p.Phe84Leu variants, and a rising prevalence of mixed phenotypes, underlining the importance of broad-based genotype–phenotype monitoring protocols (4). Also, given the non-negligible prevalence of hereditary forms even among elderly ATTR-CM patients (5.3% overall, 13% in women ≥70 years), systematic genetic testing is warranted in all cases irrespective of age (5).

In ATTRv amyloidosis, some patients primarily exhibit symptoms of polyneuropathy, traditionally referred to as familial amyloidotic polyneuropathy (FAP) or ATTR polyneuropathy (ATTR-PN); others mainly or exclusively present with symptoms of cardiomyopathy, traditionally known as familial amyloidotic cardiomyopathy (FAC) or more recently as ATTR cardiac amyloidosis (ATTR-CA). However, many patients present with a multisystemic involvement that includes sensory, motor, autonomic, and cardiac symptoms. Notably, incomplete penetrance has been observed, meaning that not all individuals carrying a pathogenic TTR mutation will develop clinical manifestations of the disease. This variability complicates both diagnosis and genetic counseling. However, once symptoms of ATTRv amyloidosis emerge, the disease often progresses rapidly, leading to severe disability and reduced life expectancy, thus, monitoring of carriers is mandatory (1).

Importantly, the genotype–phenotype association can widely vary depending on the *TTR* variant which is prognostic of disease severity and progression rate over time. Indeed, in the Emilia-Romagna Region (Italy) the most prevalent phenotype is caused by the variant p.Ile88Leu, which is characterized by early overt cardiac symptoms.

In order to provide an optimal and appropriate health care service to ATTR patients, a structured referral network was established in 2022, involving both tertiary and peripheral ATTR-CA cardiology and neurology centers. This network, referred to as the ‘ATTR Working Group,’ brings together cardiologists and neurologists from across the Emilia-Romagna Region (6). The Working Group members work both in Academic and peripheral centers and collaborate to achieve the following goals:

- To ensure that all patients in the Emilia-Romagna Region have equal access to the best possible diagnostic and therapeutic opportunities through standardized approaches to the management of ATTR patients;
- To address Regional epidemiology-related needs of patients affected by specific *TTR* variant;
- To facilitate and support scientific data dissemination.

Early diagnosis and detection of disease progression are emerging as a crucial need for ATTR amyloidosis in order to significantly impact survival, patients’ functions and quality of life (7–9). Currently, parameters to be monitored in ATTR patients in the real life might refer to some publicly available recommendations regarding the monitoring and assessment of disease progression in the real-world setting of patients with ATTRv (1, 7–9). Recent evidence suggests that early myocardial deformation abnormalities, especially reduced left and right ventricular global longitudinal strain, may be present in asymptomatic ATTRv mutation carriers despite preserved ejection fraction, and may represent early echocardiographic markers of myocardial infiltration (10). Nonetheless, a standardized disease monitoring protocol has not been established in Italy, posing a significant unmet need for a prompt and equal access to care.

Therefore, in the Emilia-Romagna Region the “ATTR Working Group” has sought to tailor the above mentioned recommendations to the Regional “real clinical setting” in order to optimize and standardize a monitoring protocol aimed at identifying disease progression. Patients’ and carriers’ access to uniform monitoring routes across the entire Region ensures optimal disease management and economic sustainability.

Moreover, ATTRv patients and *TTR* variant carriers’ data are being collected through the REDCap web platform, allowing the possibility to retrospectively assess the evolving ATTR management associated with the implementation of the Regional monitoring protocol within the referral chain.

In this article we aim to describe the ATTRv monitoring protocol that has been employed in the Emilia-Romagna Region since January 2023.

## Methods

To agree on a Regional monitoring protocol specifically tailored to the clinical needs of ATTRv patients and carriers, a specific survey of the previously identified centers (Working Group) managing ATTRv patients (6) was administered. The survey collected the monitoring methods employed at each center taking into account both the cardiological and neurological aspects related to ATTR patients (Supplementary Appendix 1). Through subsequent in-person and remote meetings, the results of the surveys highlighted the capabilities of the involved centers. Simultaneously, a review of the current cardiology guidelines and neurology recommendations available for the ATTRv setting was conducted (1, 7–9).

## Results

The Working Group identified the three most frequently observed clinical scenarios in routine medical practice and agreed on the following definitions:

1. **Asymptomatic carriers:** individuals who carry the TTR gene mutation in one allele in absence of any symptoms and signs of disease.
2. **ATTR patients:**

- **Cardiological phenotype:** patients presenting with cardiac symptoms and/or signs without neurological symptoms and/or signs.
- **Neurological/mixed phenotype:** patients presenting with exclusively neurological symptoms and/or signs, or with mixed clinical characteristics (i.e., both cardiac and neurological involvement).

### Monitoring asymptomatic carriers

According to current scientific literature, monitoring of asymptomatic carriers should begin approximately 10 years before the predicted age of disease onset (PADO) (8). In our protocol, however, monitoring is scheduled to start 15 years before PADO in order to account for potential anticipation phenomena and to minimize the risk of missing early signs of disease onset.

Specifically, asymptomatic carriers should undergo baseline clinical and neurophysiological evaluations, followed by annual assessments starting 15 years before the predicted onset of the disease in their family cluster. This recommendation is based in part on findings from a recent study by Cisneros-Barroso et al., which demonstrated that the onset of ATTRv occurs, on average, 16 years earlier in offspring compared to their parents (11).

The follow-up program includes clinical and instrumental multidisciplinary assessments to be conducted annually, biennially, or every three years (Table 1). Should one or more monitoring tests yield abnormal results, it is recommended that the patient undergo evaluation at the designated tertiary center.

**Table 1:** Assessments type and timing of carrier monitoring.

Every year	Every 2 years	Every 3 years
Electrocardiogram	24-h Holter ECG monitoring	Whole-body scintigraphy with a bone-avid tracer (in Emilia Romagna: DPD/HDP/HMDP), or cardiac MRI with gadolinium contrast.
Echocardiogram	Neurological evaluation	
Local laboratory blood tests including NT-proBNP and troponin (T-hs or I-hs)	Gastroenterology consultancy	
	Ophthalmology consultancy	
	Nephrology consultancy	

### Neurological monitoring of ATTR patients

From a neurological standpoint, the Working Group proposes a unified protocol of clinical and instrumental evaluations for both carriers and symptomatic patients, applied with different timing based on the patient category (Table 2).

**Table 2:** Neurological follow-up across the three clinical scenarios of ATTRv.

Patient category	Clinical evaluation	Neurophysiological studies
Asymptomatic Carrier	Baseline and every 2 years during the 15 years before PADO	Baseline and every 2 years during the 15 years before PADO
Cardiological phenotype	Every 6 months	Every 6 months
Neurological/mixed phenotype	Every 6 months	Annually

PADO, predicted age of disease onset.

Specifically, the working group recommends clinical evaluations every 6 months for all ATTR patients. Neurophysiological assessments should be performed annually in patients with a neurological or mixed phenotype, and every 6 months in patients with a predominantly cardiac phenotype.

This differentiated approach reflects the distinct prognostic trajectories of patients with cardiac amyloidosis (CA) and the variability in therapeutic options available for neurological versus cardiological involvement (8).

The neurological monitoring protocol includes: (1) clinical assessment, and (2) neurophysiological studies.

1. **Clinical Assessment**

Experts agree on performing a comprehensive series of clinical evaluations, which include a neurological physical examination and the use of validated self-administered scales and questionnaires, specifically:

- Complete neurological physical examination
- Measurement of weight and height for Body Mass Index (BMI, kg/m^2^) calculation
- Measurement of blood pressure (mmHg) and heart rate (bpm) in the supine position and after 3 min of standing (orthostasis)
- Application of scales selected for their relevance and feasibility in clinical practice:

**FAP/PND (Familial Amyloidotic Polyneuropathy / PolyNeuropathy Disability):** a straightforward scale used to classify patients based on ambulatory capability.
  **NIS (Neuropathy Impairment Score):** this scale ranges from 0 to 244, with higher scores indicating more severe dysfunction. It is a composite score of clinical deficits (weakness, loss of reflexes, and sensory loss) derived from the assessment of muscle strength in 24 muscle groups, reflexes in 5 groups, and sensory function in all four limbs (11).
**CADT (Compound Autonomic Dysfunction Test):** a clinician-administered scale designed to assess autonomic symptoms and signs. The total score reflects the presence and severity of orthostatic hypotension, gastrointestinal, sphincteric, and sexual dysfunction symptoms.
**R-ODS (Rasch-built Overall Disability Scale):** a 24-item self-administered questionnaire that evaluates functional ability in daily activities. It is specific to patients with peripheral neuropathy and measures the condition’s impact of on everyday tasks. Scores on this scale help identify functional impairment, assessing an individual’s ability to function independently in daily life (7).
**Norfolk QOL-DN (Norfolk Quality of Life-Diabetic Neuropathy):** a 35-item self-administered questionnaire that assesses the quality of life in patients, with a scoring range from −4 to 136 (3). This tool is designed to evaluate symptoms associated with damage to different types of nerve fibers and is structured into five domains: physical functioning/large fiber neuropathy; activities of daily living; symptoms; small fiber neuropathy; and autonomic neuropathy (12, 23).

2. **Neurophysiological Assessment**

The Working Group proposes the same neurophysiological assessment protocol for all three patient groups, with timing variations based on the clinical scenario (as outlined in Table 1).

Neurophysiological tests will be performed according to a standardized methodology:

- With surface electrodes;
- Using the antidromic technique for sensory nerve conduction studies and ortodromic for motor nerves;
- Measuring amplitude of compoud motor action potential (CMAP) and sensory nerve action potential (SNAP) from the negative to the positive peak
- Unilaterally on the clinically most affected side, or randomly (right or left) in asymptomatic carriers for the Tibial, Peroneal, Radial, nerves; Bilaterally for the Median, Sural, Dorsal Sural nerves (the and Ulnar nerve is also examined bilaterally in presence of bilateral signs of distal slowing of the sensory and/or motor conduction of the Median nerve);
- Applying normative reference values derived from available scientific evidence (13–15) for each specific test.

Reference values for each parameter measured in the individual neurophysiological tests, along with detailed information on stimulation and recording techniques, are provided in Table 3

**Table 3:** Neurophysiological tests in the follow-up of patients with ATTR.

Tests and normative value	Assembly
**Bilateral motor median** **DML:** ≤4,4 ms **MCV:** ≥49 m/s **CMAP:** ≥4,0 mV	**Stimulation**: wrist (7 cm) antebrachial **Registration**: APB
**Bilateral sensitive median** **SCV:** ≥50 m/s **SNAP:** ≥20 μV	**Stimulation**: wrist **Registration**: II finger
**Right or left motor ulnar** **DML:** ≤3,3 ms **MCV:** ≥49 m/s **CMAP:** ≥6,0 mV	**Stimulation**: wrist (7 cm) below elbow **Registration**: ADM
**Right or left sensitive ulnar** **SCV:** ≥50 ms **SNAP:** ≥10 μV	**Stimulation**: wrist **Registration**: V finger
**Right or left tibial** **DML:** ≤5,8 ms **MCV:** ≥41 m/s **CMAP:** ≥4,0 mV	**Stimulation**: ankle (9 cm) popliteal fossa **Registration**: AH
**Right or left sensitive radial** **SCV:** ≥50 ms **SNAP:** ≥15 μV **SRAR**: ≥0,21	**Stimulation**: forearm (10 cm) **Registration**: extensor tendon of the finger I
**Bilateral sural** **SCV:** ≥ 40 m/s **SNAP:** ≥6 μV	**Stimulation**: Achilles tendon beginning **Registration**: distally lateral malleolus
**Sural dorsal bilateral** **SCV**: ≥ 38.7 m/s **SNAP**: ≥ 4,5 μV **S/DNS**: ≤3,51	**Stimulation**: distally lateral malleolus (14 cm) **Registration**: IV-V finger origin
**Right or left motor peroneal** **DML:** ≤6.5 ms **MCV:** ≥44 m/s **CMAP:** ≥2.0 mV	**Stimulation**: ankle (9 cm) sub-capital **Registration**: EDB
**SSR** Present/Absent	**Stimulation**: electrical stimulation on the forehead + clap **Registration**: palm and sole four limbs

DML, distal motor latency; MCV, motor conduction velocity; CMAP, compound motor action potential; SCV, sensory conduction velocity; SNAP, sensory nerve action potential; SRAR, sural/radial amplitude ratio; S/DNS, SNAP ratio between sural and dorsal sural branch; SSR, sympathetic skin response; APB, abductor pollicis brevis; ADM, abductor digiti minimi; AH, abductor hallucis; EDB, extensor digitorum brevis.

The most suitable neurophysiological scores applicable to real practice, derived from individual measurements, should be calculated as follows:

- **Total NCS (Nerve Conduction Study):** sum of sural SNAP, ulnar SAP, tibial CMAP, and peroneal CMAP (15).
- **SNS (Sensory Neurophysiological Score):** sum of sural SNAP and median SNAP (16).
- **MNS (Motor Neurophysiological Score):** sum of peroneal CMAP and median CMAP (16).
- **Sural/DNS Ratio:** ratio of sural SNAP to SNAP of the dorsal sural nerve branch (DNS) (14).
- **SRAR (Sural/Radial Amplitude Ratio):** ratio of sural SNAP to radial SNAP (13).
- **SSRs (Sympathetic Skin Response score):** sum of lower and upper limb SSRs on the same side (16).

### Cardiological monitoring program for patients with ATTR-CA

For patients diagnosed with TTR-related cardiac amyloidosis, experts recommend cardiological evaluations every six months (i.e., cardiology visit, ECG, echocardiogram, and laboratory tests including NT-proBNP and local Troponin T-hs o I-hs), and annually (24-h Holter ECG).

In addition, it is recommended that the following assessments be performed every 6 months:

- Kansas City Cardiomyopathy Questionnaire **(KCCQ)**
- EuroQoL-5D **(EQ-5D)**
- 6-Minute Walk Test **(6MWT)**

Patients presenting with exclusively neurological involvement undergo the same evaluation protocol on a yearly basis.

The Working Group also agrees on the need for the following at the onset of specific symptoms and every 2 years afterwards:

- Nephrology consultation
- Gastroenterology consultation
- Ophthalmology consultation

## Discussion

Implementation of a regionally shared protocol for the monitoring of ATTR amyloidosis represents a relevant advancement in standardizing care and addressing both diagnostic delays and heterogeneous disease management across healthcare settings. Compared with international recommendations (1, 7–9), the monitoring protocol established by the Emilia-Romagna ATTR Working Group incorporates core principles from existing guidelines, while tailoring them to the regional clinical reality, particularly regarding accessibility and the availability of resources in peripheral centers.

From a neurological standpoint, including of both clinical scales and neurophysiological studies within a structured timeline for asymptomatic carriers and symptomatic patients reflects an effort to balance sensitivity in early detection with feasibility in daily clinical practice. While the selected scales (NIS, FAP/PND, R-ODS, CADT, Norfolk QOL-DN) are consistent with international standards, the regional protocol emphasizes routine and scheduled use of such tools, which are often underutilized in real-world settings due to logistical constraints. Moreover, standardizing of nerve conduction techniques and adopting of composite scores (e.g., SNS, MNS, SRAR, Sural/DNS) aim to improve comparability across centers and over time, an essential step toward objective disease monitoring. These methodological refinements go beyond current recommendations by proposing specific parameters and thresholds, which may serve as a benchmark for other regional systems.

In the cardiology domain, the protocol remains aligned with ESC and AHA guidelines regarding frequency and type of assessments, including echocardiography, NT-proBNP, troponin levels, and quality of life measures such as KCCQ and EQ-5D. However, the regional approach underlines the importance of integrating functional tests (6MWT) and periodic imaging (in asymptomatic carriers whole-body scintigraphy or cardiac MRI) as part of routine monitoring, even without overt clinical progression. This reflects a proactive rather than reactive strategy, supported by evidence that early identification of subclinical changes can impact prognosis and therapeutic timing. Furthermore, while most centers adopt current cardiology staging systems, the protocol guides interdisciplinary consultations (e.g., nephrology, gastroenterology, ophthalmology), anticipating multisystemic involvement.

A key strength of this initiative lies in the formalization of a shared referral chain between tertiary and peripheral centers, allowing patients and asymptomatic carriers to access the same clinical tools regardless of geographic location. This organizational framework fosters equity of care, improves early detection, and enhances the regional capacity to manage a rare and complex condition. Continuous patient-level data collection through the REDCap-based registry provides the infrastructure for prospective outcome analyses. This will allow the identification of variability in care delivery, gaps in adherence to the protocol, and opportunities for improvement, consistent with the principles of implementation science (17). Future analyses will be essential to assess whether protocol adherence correlates with improved functional outcomes, delayed disease progression, or reduced healthcare utilization.

Some limitations must be acknowledged: although the survey captured variability across centers, some differences in resources and expertise may persist, potentially affecting the uniform application of monitoring tools. Secondly, while the protocol has been designed for generalizability, its applicability to other regions or healthcare systems may require adjustments.

Nonetheless, the Emilia-Romagna ATTR monitoring protocol demonstrates that it is possible to implement a structured, multidisciplinary, and scalable disease management model even within a heterogeneous regional context. Its success will depend not only on its initial design but also on continuous quality control, training of professionals, and alignment with evolving clinical evidence and therapeutic advances.

### Emerging biomarkers and alternative monitoring strategies

While our regional protocol emphasizes standardized clinical, neurophysiological, and cardiological assessments, other innovative approaches are under investigation and may soon influence carrier monitoring strategies. Among these, cutaneous amyloid detection using minimally invasive skin punch biopsies has demonstrated promising diagnostic yield, even in pre-symptomatic stages, by identifying early amyloid deposition in peripheral small fibers (18, 19). This approach could provide an objective marker of disease onset in ATTRv carriers, potentially complementing or even anticipating changes detected by neurophysiological studies (20).

Another area of growing interest is the use of circulating biomarkers of axonal damage, particularly neurofilament light chain (NfL). Elevated plasma NfL levels have been associated with early axonal injury and correlate with disease severity in a variety of neurodegenerative and neuroinflammatory disorders (21). Recent studies in ATTRv carriers suggest that NfL may rise before overt clinical symptoms, providing a quantifiable, minimally invasive indicator of subclinical neurodegeneration (22).

Although neither cutaneous amyloid detection nor NfL assays are currently widely available in our region, their incorporation into future standardized protocols could further enhance early detection and risk stratification. By combining these emerging biomarkers with established clinical and instrumental tools, a multimodal monitoring approach may become feasible, ultimately improving personalized timing for therapeutic intervention.

## Conclusion

The multidisciplinary and multicentric ATTR Working Group project represents a response to the diagnostic and therapeutic unmet needs in managing patients and asymptomatic carriers with ATTRv. Since 2022, it has involved all hospital facilities across the Emilia-Romagna region, enabling the identification and validation of a sustainable and standardized diagnostic and care pathway. The program is tailored to the specific needs of ATTRv patients, with particular emphasis on both clinical management (care and follow-up) and the practical and psychological dimensions, including quality of life (QoL).

The ATTRv monitoring protocol is continuously evolving, whereby analysis of patients’ data collected into a web-based registry, will enhance the clinical setting of ATTRv, promote the uptake of research findings into routine healthcare and inform Healthcare providers on patient care pathway efficiency and treatment outcomes.
