# Supplementary material for: Monitoring patients and asymptomatic carriers with hereditary transthyretin amyloidosis: regional protocol of Emilia-Romagna ATTR working group
Source: Front Neurol. 2025 Aug 29;16:1666318. doi: 10.3389/fneur.2025.1666318 (PMC12426949; doi:10.3389/fneur.2025.1666318)
Supplement: Supplementary file 1 [file Data_Sheet_1.docx]

**Supplementary Appendix 1**

*Questions Directed to Cardiology (A) and Neurology (B) Specialists, Administered Through the Survey Sent to All Hospital Centers in the Emilia-Romagna Region Involved in the Project*

Abbreviations: . wtATTR: ATTR-CA: cardiac ATTR; wild-type ATTR; hATTR: hereditary ATTR;AL: Light-chain amyloidosis.

| **(A) ATTR regional referral pathway: Cardiology SURVEY** | |
| --- | --- |
| #1 | Do you have a specific clinic for the management of ATTR-CA in your hospital? If so, when was it established? |
| #2 | How long has bone scintigraphy been available? |
| #3 | How long has genetic sequencing of the TTR gene been available? |
| #4 | How long has cardiac magnetic resonance been available? |
| #5 | How many patients with cardiac amyloidosis (AL, wtATTR and hATTR) are currently managed in your clinic? |
| #6 | How many patients with cardiac amyloidosis (AL, wtATTR and hATTR) are currently managed in your clinic? (+ IRCCS S. Orsola) |
| #7 | How many patients with suspected ATTR-CA have completed the diagnostic process in your clinic ? |
| #8 | How many patients with suspected ATTR-CA have been referred to IRCCS Sant’Orsola to complete the diagnostic process? |
| #9 | How many patients with confirmed ATTR-CA have been referred to IRCCS Sant’Orsola to undergo specific treatments ? |
| #10 | How many patients with suspected ATTR-CA have been referred to a center outside of the region to complete the diagnostic process? |
| #11 | How many patients with confirmed ATTR-CA have been referred to a center outside of the region to undergo specific treatments? |

| (B) ATTR regional referral pathway: Neurology SURVEY | |
| --- | --- |
| Indicate which neurological clinical markers you consider most useful and applicable for monitoring patients under treatment, and rank them in order of importance | FAP score  PND score  SFN-SIQ  NIS  Rasch-built Over- all Disability Scale (R-ODS)  Norfolk Quality of Life (Norfolk QOL-DN)  Composite Autonomic Symptom Score-31 (COMPASS-31) questionnaire  CADT-Q  Modified Norris test (disability due to the sensory motor deficit)  10' walking test  Timed Get Up and go test |
| Indicate which cardiological clinical markers you consider most useful and applicable for monitoring patients under treatment, and rank them in order of importance | Clinical examination  NYHA class  Kansas City Cardiac Questionnaire (KCCQ)  Cardiac staging system  NAC Clinical staging system  6-Minute Walk Test (6MWT) |
| Rank the following instrumental neurological markers by order of importance for patient monitoring. | Nerve conduction studies (NCS)  Composite Nerve Conduction Score  Sudomotor testing (Sudoscan)  Quantitative Sensory Testing (QST)  Heart rate variability with deep breathing test  Orthostatic vital signs  Valsalva maneuver  Handgrip strength test (Jamar both hands grip strength test) |
| Rank the following instrumental cardiological markers by order of importance for patient monitoring. | 12‐lead ECG  LV measures wall thickness/mass  Systolic function measurements  Diastolic dysfunction worsening,  Holter ECG  electrophysiologic study (EPS) |
| Rank the following laboratory markers by order of importance for patient monitoring | NT-proBNP  Troponin (high-sensitivity) assay  Proteinuria  Albuminuria/microalbuminuria  Serum creatinine/calculated GFR  Hemoglobin  Alkaline phosphatase |

**Legenda:** FAP: ; PND: ; SFN-SIQ: Small-fiber neuropathy and symptom inventory questionnaire; NIS: ; CADT-Q: Compound Autonomic Dysfunction Test Questionnaire; NYHA: ; ECG: electrocardiogram; NT-proBNP: ; GFR: .
